# Class and Health Inequality in Later Life: Patterns, Mechanisms and Implications for Policy

**DOI:** 10.3390/ijerph14121533

**Published:** 2017-12-08

**Authors:** James Nazroo

**Affiliations:** Manchester Institute for Collaborative Research on Ageing and Sociology, School of Social Sciences, University of Manchester, Manchester M13 9PL, UK; james.nazroo@manchester.ac.uk; Tel.: +44-161-275-2495

**Keywords:** later life, inequality, health, wellbeing, class, retirement, social participation

## Abstract

The growth of the post-retirement population, which has occurred as a result of rapid growth in life expectancy coupled with the ageing of the baby boomer cohort, has led to significant concern. This concern, however, typically neglects the heterogeneity of later life experiences and how these are patterned by inequalities that reflect how process of social stratification continue to operate into later life. This paper draws on a programme of work, based on analysis of the English Longitudinal Study of Ageing, to empirically examine questions of inequality in later life. It begins by illustrating the patterning of health inequality. It then investigates the importance of later life contexts and events in shaping inequality through and after the retirement process. In doing so it examines the extent to which later life continues to reflect stable social structures that shape inequalities and, consequently, health and wellbeing in later life. The paper then illustrates how the effects of socioeconomic position on health in later life can be theorised as a product of class processes, borrowing in part from Bourdieu. Other dimensions of inequality, such as gender, ethnicity, area and sexuality, are not discussed here. The paper concludes with a discussion of the need for a close focus on inequalities in later life in research, policy and practice.

## 1. Introduction

The challenges that arise as a result of the rapid ageing of the population have been the focus of a significant body of policy and academic analysis, and of enquiries held by international and national bodies. The first International Plan of Action on Ageing was agreed in Vienna in 1982 [1], and over the last three and a half decades there has been an exponential increase in such publications. These reports have largely taken on an apocalyptic tone, even when this has been paralleled with discussion of the potential opportunities that ageing populations provide. For example, in 1999 a brief report from the early work of The Commission on Global Ageing [2] stated that:

“Among the many trends that compete for the attention of policy makers these days, none is more likely to shape economic, social, and political developments in the early twenty first century than the simultaneous aging of Japan, Europe, and the United States ... The human life cycle is undergoing unprecedented change. To preserve economic security, we must adapt the social institutions built around it to these new realities”.

This type of analysis proposes that the combined effects of the ageing of the baby boomer cohort, increased life expectancy, and reduced fertility, means that developed countries are facing a rapidly growing population of those of pension age relative to those of working age, placing significant strain on public expenditure as periods of retirement, economic inactivity and dependency lengthen. Consequently, the major strategy underpinning policy responses to ageing populations is to extend working lives and to make pension systems and the coverage of social and health care less generous. Longer periods of employment, leading to greater pension contributions and shorter periods of retirement, are proposed to provide potential solutions to such problems.

One means of encouraging longer working lives is to increase the age at which the state pension is received; a policy change that is occurring across most developed countries and one that is having some effect. For example, data from England indicate that in 2002/2003 47.5% of men aged 60–64 were in paid employment, while in 2014/2015 this figure was 58.8%; and for men aged 65–69 the figures changes even more dramatically from 15.9% in 2002/2003 and 27.2% in 2014/2015 [3]. Similar differences were present for women: in 2002/2003 61.1% of those aged 55–59 were in paid employment compared with 66.7% in 2014/2015; while the figures for those aged 60–64 were 29.7% in 2002/2003 and 42.6% in 2014/2015 [3]. Although showing a large rise in employment rates in the ages leading up to and following the notional retirement ages of 65 and 60 for men and women respectively, a substantial proportion of the population were still leaving the workforce before these ages. If attempts to extend working lives are to be more successful there needs to be a more complete understanding of the characteristics of the older workforce, the barriers to remaining in paid work, and the factors that might encourage older people to remain in employment. 

In addition to placing a focus on delaying retirement and, indeed, considering the post-retirement period as an inevitable withdrawal from society, some academic and policy work has also drawn on the idea that the end of the working life opens opportunities for engagement in socially productive and meaningful activity. This would mean that the ageing of the population gives the possibility of greater numbers of older people involved in such activities. This notion of productive, or active, ageing points to the contributions that older people can make to society through civic, social and cultural participation. In addition, this might also lead to better social and mental wellbeing for older people [4,5,6], following the suggestion that continued meaningful social engagement is a crucial component of healthy ageing [7].

Central to the challenges of maximising older people’s participation in paid employment and of developing and promoting opportunities for older people to participate more fully in society, are changes in health with age. Health has an obvious relevance as a resource, at both an individual and a societal level, for continuing involvement in paid work and productive engagement in other activities. A focus on health, though, requires a shift from an interest in just life expectancy to an interest in healthy life expectancy—not just how long people live, but also how long they remain healthy. Crucially important also is how health, life expectancy and healthy life expectancy vary across the population and the implications of this distribution for promoting economic and social participation. 

However, the distribution of health across the population, and inequalities in this, have not been a primary focus of either policy or academic work concerned with later life. For example, the framework and focus offered by the most recent UK review of inequalities in health, “Fair Society and Healthy Lives”, commonly known as the Marmot Review [8], is almost exclusively on interventions in early life. In terms of causal mechanisms, The Marmot Review emphasised those relating to early life, with some emphasis on working life and the neighbourhood environment, but with no consideration of circumstances in later life. This is reflected in the discussion of policy objectives and recommendations, which also contains no mention of later life, beyond very brief reference to “flexibility in employment and retirement”.

In this context, the focus of this paper is on describing socioeconomic inequalities in health, understanding the complex and multidimensional causal processes that lead to observed inequalities, and the implications of this for policy development. 

## 2. Materials and Methods

The findings reported in this paper are drawn from a range of analyses. The methods used are described in brief below, with full details available in the cited publications.

### 2.1. Data

The analyses use data from the English Longitudinal Study of Ageing (ELSA), which is a nationally representative panel study of men and women aged 50 or older living in private households in England [9]. The sample was initially drawn from sample members from the Health Survey for England. The sample members have been interviewed face to face and asked to complete a self-completion questionnaire biennially. The first wave of ELSA (2002–2003) had a response rate of 67% and sampled over 12,000 men and women. At the time of writing, eight waves of data have been collected.

In addition to providing panel data, where the same individuals are observed over time, an important strength of ELSA is its multidisciplinary content. In broad terms the data collected cover: demographics; economics (income, wealth, pensions, employment and consumption); physical health (a range of conditions and symptoms); physical functioning (activities of daily living, instrumental activities of daily living, and mobility); cognitive function; mental health and wellbeing; participation in social, civic and cultural activities; and social networks. In addition, every four years the study conducts a collection of biomarker data, including anthropometry, physical performance measures, blood sampling (primarily used for an assessment of inflammatory markers and analysis of genetic material), and assessment of hypertension and lung function. These design elements mean that ELSA provides a data resource that can be used to explore the complex pathways that might be hypothesised to lead to inequalities in health and wellbeing in later life.

### 2.2. Measures of Health and Wellbeing

The analyses summarised in this paper incorporate a wide range of health and wellbeing outcomes. Mortality is recorded through linkage between the study participants and death records. A second outcome considered here is frailty, which is argued to be a non-specific state reflecting age-related declines in multiple systems. As such, an assessment of frailty provides an indication of an individual’s capacity for independent living and the risk of suffering a future adverse event, such as falls, institutionalisation and mortality. To reflect this non-specific state, frailty is assessed using a measure known as the frailty index that sums the number of symptoms a person has from among a large pre-specified list [10,11]. Depression is measured using an eight-item version of the Center for Epidemiologic Studies Depression scale (CES-D) [12], which asks respondents to state whether they have experienced eight symptoms of depression within the past week. Self-rated health is measured using responses to the question of how the individual rates their overall general health, with the five options for response of excellent to poor. Cognitive function is measured using a combined executive and memory function scale [13]. Quality of life is measured using the Control, Autonomy, Self-realisation and Pleasure (CASP)-19 score, which is a 19-item measure specifically designed for use among older populations [14]. Life satisfaction is measured using the Diener five-item scale [15]. The analyses presented include, therefore, more objective outcomes (such as mortality and measured cognitive function) and more subjective outcomes (such as depressed mood and self-reported health), with some in the middle (such as frailty, which includes reports of diagnoses and functional limitations). 

### 2.3. Measures of Socioeconomic Inequalities

To measure socioeconomic position the analyses presented here largely use a measure of wealth—total non-pension wealth. This includes all of the wealth held by a household in financial assets, property, other physical assets and the assets of any business they own, and is measured net of any outstanding debts [16]. In addition, some of the models make use of other measures of socioeconomic position. Current or, for those who are no longer employed, last job is used to assess occupational class, based on the National Statistics Socio-Economic Classification, with the categories of: managers and professionals; intermediate; and routine [17]. Measures of employment, work quality and retirement are discussed where they are referred to in the following text. One of the analyses also uses a measure of subjective (perceived) social status, assessed using the MacArthur scale that asks respondents to place themselves in a position on a ten-rung ladder that reflects their social standing relative to others in relation to education, employment and income [18].

Finally, the analyses use measures of social engagement [19]. Items included here are: attendance at committee meetings for a membership organisation, club or society, including political and campaigning organisations; volunteering; membership of a religious group; membership of a social, education, arts or music group, sports club, gym or exercise class; going to the cinema, art gallery, museum, theatre, concert or opera; and meeting children, family members or friends. As well as providing an overall measure of social engagement, these items are also used to construct measures of engagement in specific domains, covering civic participation, leisure activities, cultural engagement and social networks [19].

### 2.4. Statistical Methods

A range of standard statistical methods are used in the analyses reported here. These include survival analyses, Cox proportional hazards, multilevel growth models, logistic and linear regression (typically within a longitudinal framework to model change over time), propensity score matching, and path analysis, which is an extension of multiple regression with model dependencies following those proposed in a hypothesised model (the arrows in a diagrammatic representation of the model). The method used is described for each section of the results, and references to a full description of the methods used are provided.

### 2.5. Ethics

All procedures carried out during ELSA were in accordance with the ethical standards of the institutional and/or national research committee and with the 1975 Helsinki declaration and its later amendments. The most recent ethical approval was granted by the National Research Ethics Service (NRES) Committee South Central-Berkshire, 21 December 2016, reference number 15/SC/0526.

## 3. Results

### 3.1. The Extent of Socioeconomic Inequalities in Health in Later Life

**Figure 1:**
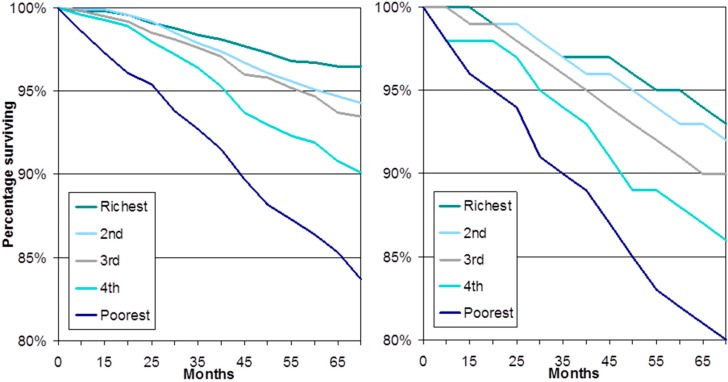
Survival rates stratified by wealth quintile: women and men aged 50 or older.

Socioeconomic inequalities in health have been systematically studied and over the past forty years there have been three major national inquires in the UK (the most recent being the 2011 ”Fair Society and Healthy Lives” review of inequalities in health led by Professor Sir Michael Marmot) [8], as well as many smaller reviews. Within this large academic and policy literature, until recently there has been almost no attention placed on describing inequalities in later life. The more recent growth in relevant literature has, however, provided strong evidence on the relationship between socioeconomic position and health in later life. Cross-sectional descriptions of the population aged 65 and older show the inverse relationship between markers of socioeconomic position, such as wealth and occupational class, and a range of markers of health [20,21,22]. Although the strength of the relationship reduces with age, this reduction appears to be largely a consequence of higher mortality rates among the most vulnerable in less affluent socioeconomic groups, with consequent reduced socioeconomic differences among survivors [23]. So, longitudinal evidence examining onset of illness and mortality among older people who were initially healthy shows marked increases in estimates of socioeconomic inequalities when mortality is considered as an outcome alongside onset of morbidity [23]. Of course, the variation in risk of mortality across population groups is an important marker of inequalities, and the availability of panel data, where the same individuals are observed over time, has allowed for an examination of these inequalities. Figure 1 shows one example of this, survival curves for men and women aged 50 or older over a six-year period stratified by wealth quintile. The level of inequality is clear, around four percent of women in the most affluent quintile do not survive over this six-year period compared with 16% of women in the least affluent quintile. Similarly, only seven percent of men in the most affluent quintile do not survive compared with 20% of men in the least affluent quintile. In this analysis a Cox proportional hazards model that adjusts for a range of health, behavioural, and, importantly, other socioeconomic factors, the hazard ratio for the least affluent quintile compared with the most affluent quintile is 1.56, that is a more than 50% greater risk of mortality once factors such as education, occupation and health behaviours have been adjusted for [22].

A concern related to these marked socioeconomic inequalities is the important question of whether alongside the well document increases in life expectancy we are seeing improvements in levels of health. Recent evidence suggests that this is not the case and, at best, at a given age in later life more recent cohorts have the same levels of frailty as earlier cohorts [11]. Alongside this, there is evidence that for the poorer segments of the population levels of frailty are higher at a given age for more recent cohorts than for earlier cohorts, suggesting an expansion, rather than a compression, of morbidity for those who are poorer [11].

**Figure 2:**
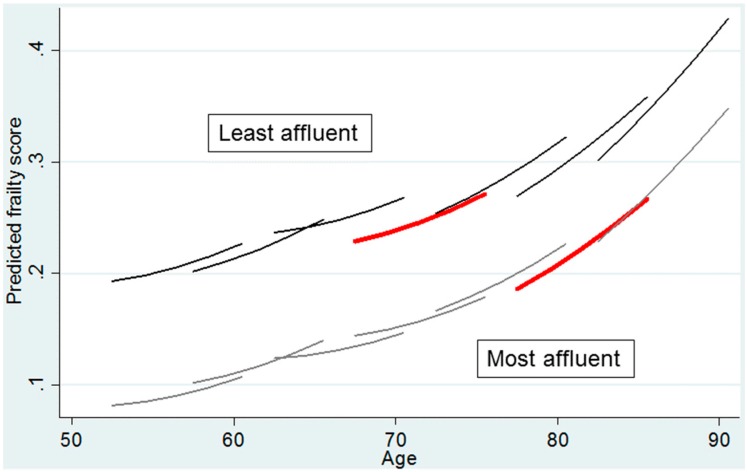
Frailty trajectories stratified by wealth tertile and cohort.

This is illustrated in Figure 2. Each line in the figures represents the change in the mean level of frailty, estimated using multilevel growth modelling of the frailty index, for a five-year cohort over an eight-year period, from 2002 until 2010—a frailty trajectory [11]. This is stratified by wealth (those in the most affluent tertile of the population compared with those in the least affluent tertile). As each line covers an eight-year period, the analysis allows for levels of frailty at a given age to be compared across age cohorts (the point of overlap on the x-axis between lines). The wealth differences in levels of frailty are stark; the trajectory of frailty for an individual in the most affluent tertile is comparable to that for those ten or more years younger in the least affluent tertile—compare, for example, the two red lines. The figure also suggests that inequalities in levels of frailty are widening—the gap between the frailty trajectories for the wealthiest and least wealthy categories are wider at younger compared with older ages. Perhaps most troubling is that among the least affluent tertile more recent cohorts appear to have higher levels of frailty compared with earlier cohorts. Take, for example, levels of frailty between the ages of 75 and 80 for the two cohorts that cross this age range. In contrast, for the most affluent tertile there are few differences in frailty across cohorts. A key driver of this wealth-specific cohort difference is the slower estimated growth rate of frailty for those in the most affluent tertile compared with the least affluent tertile [11].

This possible phenomenon, that inequalities in health in later life are increasing across cohorts and that healthy life expectancy might be worsening for poorer segments of the population, is a cause for concern. The reasons behind these changes are not clear. They could be a consequence of widening inequalities in socioeconomic position, or a reflection of the success of medicine, with those in ill-health living longer than they used to and this being particularly the case for those in less affluent socioeconomic groups, or a consequence of higher levels of illness as a result of higher levels of socioeconomically determined behavioural risk factors, such as obesity. They do, however, point to the need for a thorough investigation of causal processes, careful policy development and evaluation of interventions. This paper now turns to an examination of causal processes.

### 3.2. The Impact of Inequalities in Later Life Transitions on Health and Wellbeing

In the context of the large body of inequalities in health research that has adopted a life course focus, and largely emphasised the importance of early life, it is worth providing a corrective focus on the impact of how the transitions experienced in later life may influence inequalities in later life. The recent body of research that has focussed on a concept of wellbeing provides a route into understanding the importance of later life transitions. Wellbeing is, of course, a multi-dimensional concept [24], with many of those working in the field conceptualising it in terms of two dimensions: eudemonic (typically captured as satisfaction with life and as self-actualisation) and hedonic (typically captured in relation to positive and negative mood). Eudemonic wellbeing places emphasises on cognitive judgements of one’s life theorised in relation to autonomy and self-actualisation [25,26]. Hedonic wellbeing, on the other hand, emphasises mood driven by evaluative, rather than cognitive, judgements of one’s life [27]. Both the evaluative and cognitive aspects of wellbeing are strongly subject to processes of adaptation and changing goals, in line with theories of selective optimisation and compensation in later life, so, consequently may change, but change differently, with ageing [28]. So, as expectations change into later life, subjective evaluative appreciations of quality of life may become relatively more positive even though objective cognitive appraisals may judge circumstances as worse [24]. 

**Figure 3:**
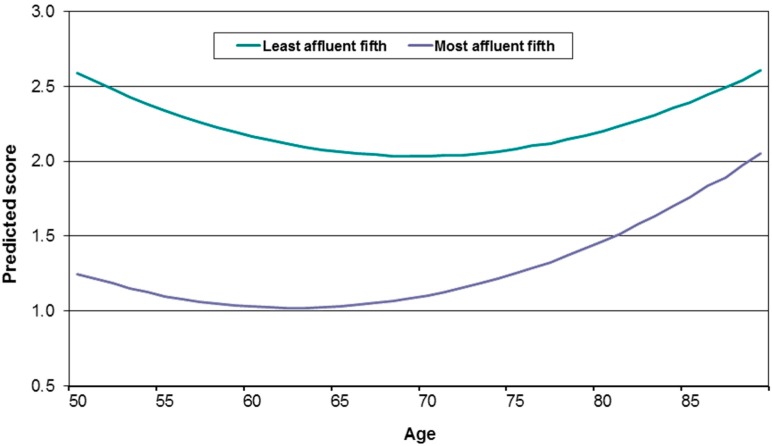
Age and level of depressive symptomatology, stratified by wealth.

Nevertheless, almost all research in this field has noted an inverted U-shaped relationship between each of these approaches to conceptualising wellbeing and age, with wellbeing improving from the early 50s to the late 60s, and then beginning to decline [29]. Importantly, across the age range that defines this inverted U-shaped wellbeing outcome, there are marked socioeconomic inequalities, as indicated in Figure 3 [30]. Here there is the expected U-shaped relationship with the negative outcome of depressed mood, but regardless of this change across ages the level of depressed mood for those in the least affluent wealth quintile remains consistently much higher than that for those in the most affluent wealth quintile. Indeed, at none of these ages do the levels of depressed mood in the least affluent wealth quintile drop to the highest level found for the most affluent wealth quintile—the levels of depressed mood for the least and most affluent quintiles of the population just do not overlap. Also of interest is that once the age-varying characteristics of health and marital status (primarily becoming a widow(er)) are taken into account, the decline in wellbeing in later life disappears. Those whose marital status does not change and who remain healthy experience ongoing improvements in their wellbeing. Of course, following the socioeconomic inequalities in health approach, deteriorating health and widow(er)hood are not random events. Rather they are events that are more likely to be experienced by those who are less affluent. The implication, then, is that socioeconomic circumstances shape the experiences of wellbeing in later life, as well as those of health.

The inverted U-shaped relationship between wellbeing and age, which suggests that important changes are occurring across the ages of 60 to 70, raises the question of what the impact of work and retirement is on health and wellbeing, and what determines these relationships; a question that is also relevant to the policy focus on extended working lives.

Recent studies provide a mixed set of findings on the relationship between health and later life work. Some show that continued working is beneficial [31,32,33,34], while others find it to be detrimental to health and wellbeing [34], or show no significant relationship [33,35,36]. One reason for the variation in findings across these studies might be that the contexts of research vary and that there are variations in the kind of paid work studied. In fact, other evidence suggests that the quality of the paid work and the type of occupation engaged in play a role in determining the health and wellbeing of older employees, with better quality and sedentary work associated with better health [37,38] and lower quality and manual work associated with poorer health [39].

**Figure 4:**
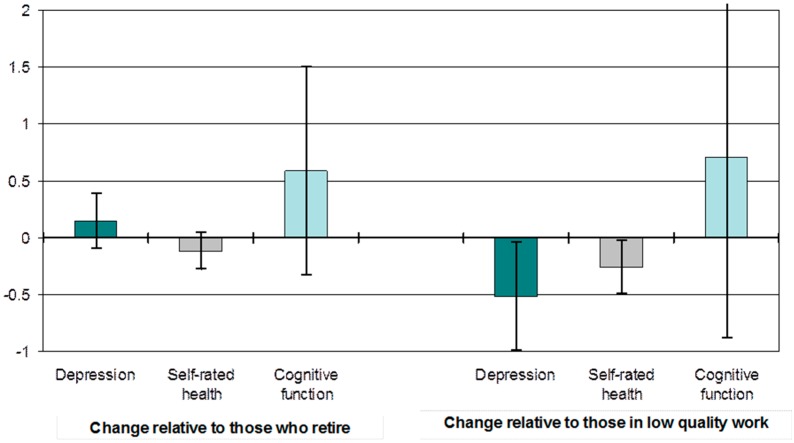
Impact of retirement on change in health: all who stay in work post state pension age compared with those who retire; and those who stay working in high quality work compared with those who stay working in low quality work.

The impact of working post state pension age on health and wellbeing is shown on the left-hand side of Figure 4 [13]. This uses a statistical technique known as propensity score matching that ”corrects” for bias in the likelihood of working post state pension age by matching workers with non-workers on the factors that relate to working, thereby producing an unbiased assessment of the differences in outcome [40]. This part of Figure 4 directly contrasts changes in health (depressed mood, self-rated health and cognitive function) for those people who worked beyond the traditional UK State Pension Age (65 for men and 60 for women) with those who worked until reaching that age and then took retirement. The height of the bars shows the difference in the health outcome (for those who continue to work relative to those who retire, who are represented by the value ”0”), with the ”I” bars showing 95% confidence intervals for these estimates. Differences are not large nor statistically significant, which suggests that any differences that might be observed between the two groups at a descriptive level are not a result of the experience of working itself, but rather the result of factors that increase the propensity of individuals to work, such as level of wealth, social class and education, partner’s employment status, level and type of pension entitlement, etc. [13].

The right-hand side of Figure 4 refines this analysis by examining differences in the quality of the work experience among those who continue to work. It does this by contrasting those who are in “poor” quality work, where the worker reports feeling poorly-reciprocated in terms of adequate salary, security, support and prospects, with those in “good” quality work, where the worker feels well-reciprocated in relation to these factors. An effort-reward imbalance scale is used to measure this concept [41]. The findings presented in the figure, again using the propensity score matching technique, show that those who continue to work in high quality employment, compared with those in poor quality work, experience a relative reduction in depression scores and improvement in self rated health over time, and an improvement in cognitive function, although the difference for cognitive function is not statistically significant [13]. 

**Figure 5:**
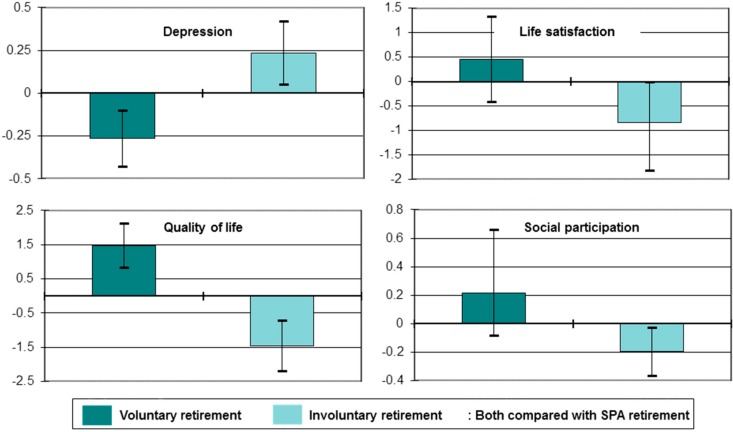
Impact of retirement type on change in health and wellbeing.

One implication of these findings is that for those working in poor conditions retirement may lead to a relative improvement in health and, indeed, there is some evidence in support of this proposition [42]. However, the on-average influence of retirement on health is neutral [13], suggesting the need to consider the complex and varied nature of the retirement process when considering its implications for health and wellbeing post-retirement. Figure 5 does this by contrasting changes in four quality of life outcomes for those who take “routine”, “involuntary” or “voluntary” routes into retirement. “Routine” retirement includes those who said that they retired because they reached retirement age; “involuntary” retirement applies to those who retired for reasons beyond their control (such as the onset of ill health, the ill health of a family member or friend, redundancy or unemployment and subsequent inability to find another job); while “voluntary” retirement includes those who retired in order to spend more time with family, enjoy life while still young and physically able, experience change from a job of which they were bored, to give the younger generation a chance in employment, to retire at the same time as their partner or spouse, or because they were offered reasonable financial terms. 

In fully adjusted longitudinal linear regression models, compared with routine retirement, involuntary retirement was associated with a worsening of depressed mood, life satisfaction, quality of life and social participation [13]. In contrast, compared with routine retirement voluntary retirement was associated with an improvement in depressed mood and quality of life (with improvements in life satisfaction and social participation not statistically significant). 

In sum, these analyses suggest that retirement is only beneficial when the pathways into retirement and the circumstances within it are optimal. Similarly, working in later life, as opposed to retiring, may be beneficial to health as long as it is good quality work, but it may be detrimental when working conditions are not ideal. As lower levels of income and less advantaged class positions are likely to be associated with participation in poorer quality work, as well as with poorer retirement circumstances, it seems particularly important to focus on the wellbeing of those in such circumstances.

### 3.3. The Significance of Class in Later Life

As indicated, socioeconomic inequalities and the nature of transitions in later life, including those related to work, might be considered to reflect broader class processes. Most research examining the role of class in generating health inequalities has followed a tradition based on a conception of class as labour relations, operationalised through measures of occupational class [43,44,45]. However, occupational class is likely to be less theoretically robust in defining life chances, or reflecting class position, once people retire from paid employment, or consider themselves to be retired. And similar problems also exist when inequalities are examined in relation to education (with an implicit focus on the significance of early life) and income or wealth (with an implicit focus on material conditions). Indeed, retired people have been excluded from much class analysis, including that exploring health inequalities [46,47,48,49,50,51,52], although there are some exceptions [23,53,54,55,56]. Consequently, it is worth considering how class might be conceptualised and how it operates in later life, and what this means for empirical investigations of its relationship with health and wellbeing.

Bourdieu provides a grounding for this by arguing that status in the class structure is dependent not only on relations within labour markets, rather it also has a symbolic dimension that is related to consumption patterns, or lifestyles [57]. He drew a distinction between the occupational characteristics by which people are classified and secondary properties of class that relate to lifestyle, and argued that these secondary properties exist as economic, cultural and social capitals [58]. Economic capital comprises assets that can be directly converted into money (such as houses and stocks and shares). Cultural capital is embodied in highbrow cultural and material tastes. To access cultural capital, one must possess the means to “consume” cultural and material goods, including sufficient physical energy, knowledge and competencies. Social capital is gained through membership of social groups, personal and formal. Bourdieu argues that “durable” social networks give access to the collective capital of recognition and support [58]. 

Important, though, is that social, cultural and economic capital, may also influence health indirectly through their influence on the perception that individuals have of their social status [68]. This connects to Bourdieu’s own argument [57], where he suggests links between economic capital, cultural capital and perceived social status in his discussion of the “natural distinction” of the bourgeois. There is now extensive evidence that perceived social status is linked to health through the relatively greater experience of low grade chronic stress of those lower down the social hierarchy [69] and that this association is independent of the influence of more objective economic indicators [18,54,70,71,72].

Relating Bourdieu’s approach to the context of health inequality, wealth determines the material conditions of life, which, it is argued, have a direct impact on health, while lifestyle habits directly link cultural capital to health: “the body is the most indisputable materialization of class taste” [57]. And supportive networks may have a direct link to health through the mutual support and shared experience between members that they bring. Intensity of participation in social and civic networks is widely used as a measure of social capital [59,60,61,62,63], and there is some evidence that this is directly beneficial to health [64,65,66,67]. 

**Figure 6:**
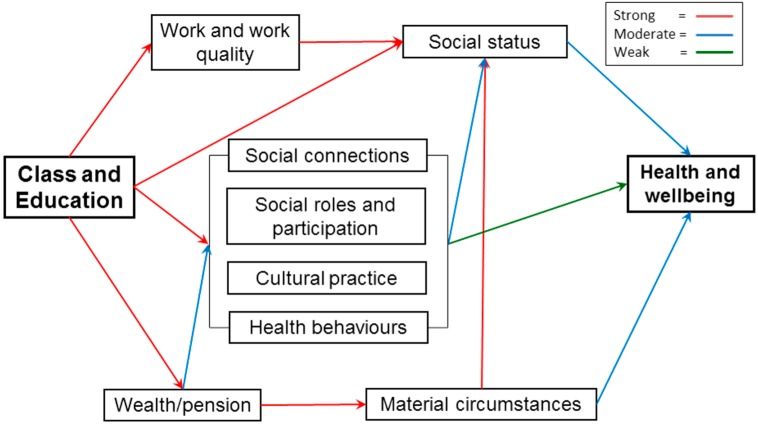
Causal mechanisms associating class with health and wellbeing in later life.

So, inequalities in health in later life might be conceptualised in terms of the economic, social and cultural resources that the older person has access to and how these relate to class position, a model strongly related to that implied by the Marmot Review. The possible mechanisms involved in this are summarised in the empirically based schematic presented in Figure 6. This is a conceptual model that proposes (with empirical testing) the relationship between inequalities in health and economic (wealth/pension, material circumstances, work and work quality), social (social connections, social roles and participation) and cultural (cultural practice and health behaviours) resources, with social class and education proposed as distal determinants, and perceived social status as a mediator. The colours of the arrows in the diagram show the outcome of the empirical testing of this conceptual model using path analysis, longitudinal data and a range of health outcomes (self-reported health, depressed mood, and a more objective assessment of activities of daily living) [73]. This shows the importance of material circumstances, employment quality, social and cultural participation, health behaviours, and the interrelationships between these factors, on change in health, and that to a certain extent these operate through their impact on perceived social status.

A central issue in Figure 6 is social, cultural and civic participation. There are a number of causal pathways through which an effect of social participation on health has been theorised, including psychological, physiological and behavioural. Social integration theory suggests that social engagement can provide a sense of purpose, which may be lacking in older age as people move into retirement and away from paid work, thereby maintaining perceived social status [74,75]. The replacement of time spent at work with more leisure-orientated activities in later life may ensure the psychological benefit of social engagement is sustained over the retirement transition. Social activity can also affect physical and mental health outcomes by enabling people to deal with emotional feelings and improving coping abilities in stressful situations [76,77,78]. Finally, social activity that requires physical exertion can improve physiological functions, such as cardiovascular health and immune function [74].

**Figure 7:**
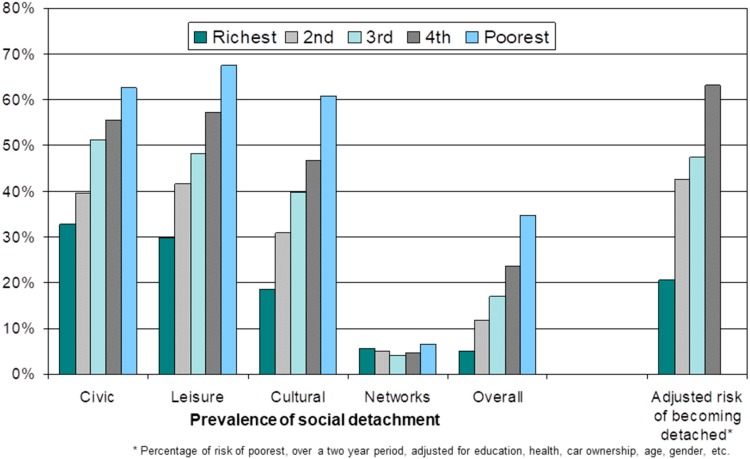
Social detachment, risk of social detachment and wealth.

In the context of an examination of class inequalities in later life, it is worth considering the ways in which markers of class relate to risk of social detachment. Evidence shows that those most likely to remain socially engaged in later life, and less likely to become socially detached, are those in a more affluent socioeconomic position and better self-rated health [79,80,81,82]. These findings suggest that social engagement is intrinsically linked with experiences across the life course that cumulatively lead to greater inequalities in later life [83,84]. Figure 7 illustrates this clearly [19]. The first five blocks of columns show that in a fully adjusted cross-sectional logistic regression model the relationship between the chance of being socially detached, overall and in four specific domains, increases the lower an individual’s level of wealth, with the one exception of the networks’ domain (a domain where overall levels of social detachment are very low). The final block of columns uses a longitudinal logistic regression model to estimate the chance of moving into social detachment compared with the least affluent wealth quintile for those in the four other wealth quintiles. It shows that this chance decreases with increasing wealth. For example, the chance for those in the richest quintile to move into social detachment is approximately 20% of the chance of those in the poorest quintile [19].

Another example can be found in relation to formal volunteering. Evidence suggests that later life volunteering on its own has a protective effect for mortality [85], self-rated health [86,87], and depression [7,86,87,88], and that it increases quality of life and life satisfaction [86,88]. These beneficial effects might, according to continuity role theory, result from the establishment of roles that substitute for paid work in terms of remaining socially productive, socially integrated and maintaining purposeful and valued roles [89], and, consequently, perceived social status. Indeed, some evidence suggests that the beneficial effects for volunteering may not be present when the role is perceived to not be rewarding [88]. Not surprisingly, following the Bourdieusian model of class inequalities in later life, outlined above, it is also the case that the opportunity to engage in volunteering activities in later life is shaped by class related economic, social and cultural capital—for example, those in the wealthiest quintile are three times more likely to volunteer than those in the poorest wealth quintile [88].

## 4. Discussion

This paper reports on research that provides clear evidence of large socioeconomic inequalities in health in later life and examines the class-related mechanisms that lead to such inequalities. It first shows that socioeconomic position, as assessed by economic wealth (a summary indicator capturing life-time material conditions, but also other characteristics that are associated with the accumulation of wealth) is strongly related to health and wellbeing outcomes. Those in the least affluent wealth quintile, compared with the most affluent quintile, have a four times higher risk of mortality over a six-year period for women, and a three times higher risk for men. This large and meaningful difference across levels of wealth is also found for age-cohort specific frailty trajectories, with those in the least affluent third of the population experiencing levels of frailty that are equivalent to those ten or more years older in the most affluent third of the population. And findings are similarly large for wellbeing, where the levels of depressed mood for the most affluent fifth of the population at age 85 or older, when they are at their highest level, remain below those for the least affluent fifth of the population at age 65–70, when they are at their lowest level. Analysis not shown in this paper also demonstrates that the decrease in wellbeing from around age 70 onwards is associated with an increased risk of poor health and death of a spouse, events that become increasingly likely the lower a person’s level of wealth [30].

In addition to examining overall age-related transitions in wellbeing, the paper also reported on research examining transitions in relation to later life work and retirement. This shows that both working post-state pension age and retirement at any age has no net effect on health or wellbeing. Rather, it is the circumstances within which work and retirement occur that are crucial. So, those who work post state-pension in good quality employment experience improvements over time in their levels of depressed mood and self-rated health when compared with those who are working in low quality work, with the suggestion of similar findings for cognitive function. And those who retire involuntarily have increases over time in their level of depressed mood and declines in their levels of life satisfaction, quality of life and social participation, when compared with those who retire at state pension age, while the opposite is true for those who retire voluntarily.

In order to examine the class-related mechanisms that might drive such inequalities in health and wellbeing in later life, the paper presents an empirical model informed by Bourdieu’s investigations of class structures [57]. The reasoning behind the adoption of this approach is that traditional markers of class, such as occupation, education or income, are both partial and less precise in a life-stage where retirement is increasingly likely. This approach tests the role of indicators of economic capital, cultural capital and social capital using a longitudinal path model, and shows how these dimensions inter-relate and lead to inequalities in health and wellbeing outcomes, in part through their impact on perceived social status. In addition, a central component of that model, the strong relationship between economic capital (as indicated by wealth) and social and cultural capitals (as indicated by different dimensions of social detachment) is demonstrated.

So, one important dimension of these findings is their illustration of the importance of class in shaping later life experiences, and the need to use a concept of class that goes beyond economic class to incorporate processes of stratification operating across social domains, such as the economy, culture and civil society [57]. This is partially reflected in the conceptual model presented in Figure 6, which points to the role of broader social and economic structures and the inequalities they generate in shaping older people’s experiences. In order to understand inequalities in health and wellbeing in later life, then, we need to come to terms with this wider nexus of material and social inequalities and to explore further these processes of stratification.

While the emphasis of this paper has been on describing socioeconomic inequalities and examining how they relate to class-processes, inequalities are also present and strong in relation to other factors, such as gender, ethnicity/race, geography, and sexuality, etc. [90,91,92,93,94,95,96]. However, there is very limited evidence to provide a nuanced description and theoretically informed discussion of underlying mechanisms related to how these factors operate in later life. It should be a priority to examine in detail the operation of these dimensions of inequality in later life.

Despite the evidence presented in this paper, as discussed earlier both interventions and broader policy work focussed on inequalities in health have had a primary focus on early life and have ignored older people and relevant processes operating in later life. The focus on early life in policy (and research) is perhaps not unexpected considering how health (and socioeconomic) inequalities are shaped across the life course. Successful interventions that are focussed on early life would address early life determinants and, more indirectly, those acting at critical periods across the life course, the accumulation of disadvantage, and adverse trajectories. Such impacts would serve to reduce inequality in later life. For example, while the research reported here highlights the importance of considering involuntary retirement and the impact of this on material circumstances and social detachment post retirement, the risk of involuntary retirement is linked with poorer working conditions, which may be linked with poorer educational outcomes and more disadvantaged childhood circumstances. In this way, involuntary retirement can be seen to be a consequence of social inequalities that may have persisted across the life course, but which then shape transitions in later life before they impact on health and wellbeing in later life. 

Nevertheless, insofar as interventions draw on evidence and recommendations provided by existing policy work, it is likely that they will ignore processes related to later life and will not have optimal impact on existing inequalities among older people. So, despite the operation of such life course effects, a focus on later life might point to additional opportunities for intervention for current generations. For example, there is growing evidence on the possibility that events and circumstances occurring at and after retirement might be relevant to both the occurrence and maintenance of health inequalities in later life. Some of this relates to the persistence of material inequalities into later life (such as levels of wealth, quality of housing, etc.), some to differences in the ways in which transitions are experienced for different socioeconomic groups (for example, involuntary versus voluntary retirement), and some to the likelihood of a negative transition occurring (for example, the relative probability of a spouse becoming seriously ill or dying).

Progress in developing an evidence base for policy development can, then, be made by focussing on circumstances/events/transitions that are particularly relevant for older people, including retirement, death of spouse and close friends, and the onset of illness and disability. How life is lived leading up to, through, and after these transitions will depend on access to and mobilisation of resources that allow the development and maintenance of social connections, networks and rewarding and valued roles, and facilitate the protection of standards of living. Unequal access to such resources has important implications for health and wellbeing inequalities in later life. It is here that it is important to have a focus on macro-social structures, those that shape how transitions are experienced and provide (or not) resources to manage transitions and consequent states.

A consideration of this suggests a need for policy development to focus on addressing social and economic inequalities in later life, pointing to the need to focus on social welfare, tax and pension reform, as well as considering area related factors such as housing, green spaces and the provision of opportunities and spaces for social, civic and cultural engagement. Disappointing in this context is that where significant reform is occurring, such as in the case of pensions and extended working lives, little consideration is given to the question of impact on economic, social and health inequalities. Indeed, reforms to increase state pension age, alongside reforms to pensions that reduce benefits and individualise risk, are likely to increase inequalities among older people. For example, the research shown here on the working circumstances of older people demonstrates that continued work is actually detrimental to the health of those in poor quality jobs, and of benefit for those in good quality jobs. This means that encouragement to work for longer periods of time could potentially result in adverse outcomes. Indeed, increasing pension ages may well force those who cannot afford to retire before receiving the state pension into longer periods of working and many of these will be people in poor quality employment. In turn, these people are placed at risk of the onset of poor health as well as exacerbation of existing (especially work-related) mental and physical health problems. Similarly, the research reported here demonstrates the importance of material circumstances in shaping health and wellbeing in later life, pointing to the need for pension reforms to be carried out in a way that considers how economic inequalities can be addressed in order to maximise the wellbeing and potential of older people. Not considered so far in this paper is the importance of equitable access to high quality health and social care, which may provide a reduction in inequalities in outcomes of care [97] and improvements in wellbeing.

## 5. Conclusions

In conclusion, this paper brings together recent evidence documenting the importance of socioeconomic inequalities in health and wellbeing in later life and how these inequalities are best understood to be a result of class-related mechanisms operating into later life. For example, those in the least affluent third of the population experience levels of frailty that are equivalent to the levels found for those who are ten or more years older in the most affluent third of the population, and they are experiencing an increase in levels of frailty in more recent cohorts, leading to a widening of these inequalities and a possible expansion of morbidity. The paper draws on Bourdieu [57] to consider class-related mechanisms for these inequalities, on the basis that more conventional approaches are less applicable in later life, and shows the importance of each of economic, cultural and social capital to these inequalities in health and wellbeing in later life, and also shows how these forms of capital relate to each other.

Despite the accumulation of evidence on these issues, policy work, whether focussed on inequalities in health or on ageing populations, has ignored processes that operate in later life to generate and maintain inequality—such as later life work, retirement, pension provision, housing, the persistence of material inequalities into later life, and access to opportunities for social, civic and cultural participation. Given the evidence, the recommendation following from this research is that the consensus on policy options needs to be rethought. Ongoing policy work in relation to ageing populations could, rather, be used as an opportunity to pay direct attention to reducing social and economic inequalities in later life in order to ensure everyone is able to live long, enjoyable and meaningful lives. Not only does this address questions of social justice, it also has the potential of reducing the strain on public and personal expenditure as the proportion of the population who are in retirement increases.
